# Functional Fcgamma Receptor Polymorphisms Are Associated with Human Allergy

**DOI:** 10.1371/journal.pone.0089196

**Published:** 2014-02-21

**Authors:** Jianming Wu, Rui Lin, Jinhai Huang, Weihua Guan, William S. Oetting, P. Sriramarao, Malcolm N. Blumenthal

**Affiliations:** 1 Department of Veterinary and Biomedical Sciences, University of Minnesota, St. Paul, Minnesota, United States of America; 2 Department of Medicine, University of Minnesota, Minneapolis, Minnesota, United States of America; 3 Department of Biostatistics, University of Minnesota, Minneapolis, Minnesota, United States of America; 4 Department of Experimental and Clinical Pharmacology, University of Minnesota, Minneapolis, Minnesota, United States of America; Cincinnati Children’s Hospital Medical center, United States of America

## Abstract

**Objective:**

IgG Fc receptors (FcγRs) play important roles in immune responses. It is not clear whether FcγR receptors play a role in human asthma and allergy. The aim of current study was to investigate whether functional single nucleotide polymorphisms (SNPs) of FcγR genes (*FCGR*) are associated with human asthma and allergy.

**Methods:**

Functional SNPs of *FCGR2A* (FcγRIIA-131His>Arg, rs1801274), *FCGR2B* (FcγRIIB-187Ile>Thr, rs1050501), *FCGR2C (*FcγRIIC-13Gln>Stop, rs10917661), *FCGR3A* (FcγRIIIA-158Val>Phe, rs396991), and *FCGR3B* variants (FcγRIIIB NA1 and NA2) were genotyped in an asthma family cohort including 370 atopy positive, 239 atopy negative, and 169 asthma positive subjects. The genotype and phenotype data (asthma, bronchial hyper-responsiveness, and atopy) of subjects were analyzed using family-based association tests (FBAT) and logistic regression adjusted for age and sex.

**Result:**

The FcγRIIA-131His>Arg SNP is significantly associated with atopy in a family-based association test (*P* = 0.00287) and in a logistic regression analysis (*P* = 0.0269, OR 0.732, 95% CI: 0.555–0.965). The FcγRIIA-131His (or rs1801274-A) allele capable of binding human IgG2 has a protective role against atopy. In addition, the rare FcγRIIB-187Thr (or rs1050501-C) allele defective for the receptor-mediated inhibitory signals is a risk factor for atopy (*P* = 0.0031, OR 1.758, 95% CI: 1.209–2.556) and IgE production (*P*<0.001). However, variants of activating FcγRIIIA (rs396991), and FcγRIIIB (NA1 and NA2), and FcγRIIC (rs10917661) are not associated with asthma, BHR, and atopy (*P*>0.05).

**Conclusions:**

FcγRIIA and FcγRIIB functional polymorphisms may have a role in the pathogenesis of allergy.

## Introduction

Asthma is a complex syndrome characterized by airflow obstruction, bronchial hyper-responsiveness (BHR), and airway inflammation. Both genetic and environmental factors contribute to the development of asthma. Evidence for a genetic component in asthma includes familial clustering and higher concordance rates in monozygotic twins than in dizygotic twins [1], [2]. Approximately 48–79% of asthma risk is attributable to genetic factors [1], [2]. According to the American Academy of Allergy, Asthma and Immunology, half of the 20 million Americans with asthma have allergic asthma. Thus, allergic reactions to foreign antigens are considered as the most common causes for asthma. To date, no genes have been definitely shown to influence asthma/allergy development. It is well-known that IgE and its cognate receptor (FcεRI) are important mediators in allergic reactions [3]. However, the role of human IgG Fc receptors (FcγRs) in asthma and allergy remains unknown.

A recent meta-analysis of human genome-wide association study (GWAS) revealed a significant asthma susceptibility locus on chromosome 1q23, where FcγR (*FCGR*) genes are located [4]. Human FcγRs are glycoproteins that bind the Fc region of immunoglobulin G (IgG). FcγRs mediate a variety of immune functions such as antigen presentation, immune complex clearance, phagocytosis of pathogens, degranulations, ADCC, and cytokine production [5]. In humans, five genes (*FCGR2A, FCGR2B, FCGR2C, FCGR3A,* and *FCGR3B*) in the 1q23 chromosome region code for five classical low affinity Fcγ receptors (FcγRIIA, FcγRIIB, FcγRIIC, FcγRIIIA, and FcγRIIIB). Coordination between the activating FcγRs (FcγRIIA, FcγRIIC, FcγRIIIA, and FcγRIIIB) and the inhibitory FcγR (FcγRIIB) is crucial in balancing immune responses and determining the outcomes of local and systemic inflammations [6]. FcγRs have important roles in the pathogenesis of a variety of human inflammatory diseases [7]. Not surprisingly, functional polymorphisms of FcγR have robust effects on susceptibility and severity of inflammatory diseases as demonstrated in genetic association studies by our group and others [8], [9], [10], [11]. However, comprehensive genetic analysis of human *FCGR* genes in asthma/allergy patients has yet been performed. It remains unknown whether human FcγRs play a role in the development of allergy.

## Patients and Methods

### Study Subjects

Genomic DNA was isolated from anti-coagulated peripheral blood of human subjects from 27 multigenerational families with multiple asthmatic members, which were originally recruited as part of the Collaborative Study on the Genetics of Asthma (CSGA) [12]. For the CSGA, asthma families were ascertained through two asthmatic siblings. Additional relatives in the families were then recruited either by extending the families through asthmatic relatives or by including no more than one unaffected relative to permit a lineage to incorporate other relatives with asthma. The inclusion criteria for each family consisted of each of the two asthmatic siblings having met the following criteria for the proband: (1) being at least 6 years of age; (2) having either bronchial hyper-responsiveness (BHR), defined as a fall from baseline FEV1 greater than 20% in one second after inhalation of 25 mg/ml or less of methacholine, or reversibility, defined as a 15% or greater increase from baseline FEV1 after inhaled bronchodilator (albuterol) for those with reduced baseline FEV1; (3) having the presence of two or more of the symptoms of coughing, wheezing and shortness of breath; (4) having less than three pack/years of cigarette smoking; and (5) having a physician’s diagnosis of asthma with no conflicting pulmonary disease. All family members went through a standardized protocol consisting of an interviewer administered questionnaire, pulmonary function studies including a methacholine challenge and/or reversibility studies, blood drawing for serum IgE levels at a single time not during an acute exacerbation and skin prick testing using standardized allergens [12]. Additional details of the study design can be found in an earlier publication [12]. The 27 multigenerational Caucasian families were recruited in Minnesota as previously described [13]. These families had 169 asthmatic members, 347 who were not asthmatic and 129 for whom the diagnosis was unavailable [13]. Pulmonary function data were available on 619 individuals. The study (Title: Genetics of Asthma. Study Number: 920M05150) was approved by The Institutional Review Board of Human Study at the University Of Minnesota. The informed written consent was obtained from all participants recruited in this study. The written consents containing participants’ signatures were kept in locked file cabinets for record. The traits of asthma, BHR, atopy, and IgE levels were analyzed in the current genetic study.

### Genotyping of *FCGR* SNPs

*FCGR* family member genes were generated through duplication and divergence during evolution [14]. SNPs in five *FCGR* genes are not suitable for direct TaqMan assays due to near 100% sequence identity surrounding the functional SNPs between homologous genes. Consequently, we used a modified *FCGR* SNP TaqMan assay in which *FCGR* gene-specific PCR fragments were used as templates instead of genomic DNA for TaqMan assays. The genomic DNA fragments containing functional SNPs of *FCGR2A* and *FCGR3A* were amplified using the gene specific primers as described previously [9]. For the *FCGR2B* SNP, a genomic DNA fragment containing FcγRIIB-187Ile>Thr was amplified using the gene specific primers as described [8], [15]. To genotype FcγRIIC*-*13Gln>STP, a long *FCGR2C* genomic fragment (6227 bps) containing the SNP was amplified using Platinum Taq DNA Polymerase High Fidelity (Invitrogen) with a sense primer (5′-CTG CAT ATG TTG TCC CCC TGT GTT GCT AAA T-3′) annealing to the *FCGR2C* intron 2 and an antisense primer (5′-AAC ATG AGA GAG AAA AAG AGA GGC AGG GAG GGA GCT TA-3′) annealing to the *FCGR2C* intron 6. The TaqMan assays for *FCGR2A* SNP (FcγRIIA-131His>Arg), *FCGR2B* SNP (FcγRIIB-187Ile>Thr), *FCGR2C* SNP (FcγRIIC-13Gln>STP), and *FCGR3A* SNP (FcγRIIIA-158Val>Phe) were designed using the Software Primer Express v3.0 (Applied Biosystems Inc.). TaqMan genotyping assays were carried out according to the standard protocol on an ABI 7500 Real-Time PCR System using Genotyping Master Mix (Applied Biosystems). The primers and probes used in *FCGR* TaqMan genotyping assays are listed in Table 1. Genotyping of the respective SNPs of *FCGR2A*, *FCGR2B*, *FCGR2C*, and *FCGR3A* was carried out with four independent TaqMan allele discrimination assays that were developed and validated in the lab. The specificity and accuracy of individual TaqMan assays were validated by the perfect match (100%) with at least 300 genotyped human subjects published previously [8], [9], [15]. For *FCGR3B* allele determination, a primer pair that specifically amplifies the *FCGR3B* fragment containing *FCGR3B* coding SNPs (cSNPs) was used. The 1.6 kb *FCGR3B* PCR fragment was treated with ExoSAP-IT PCR Product Clean-UP reagent (Affymetrix) before being sequenced on an ABI 3730xl DNA Analyzer with BigDye Terminator kit (Applied Biosystems) with the sequencing primer (5′-TCC TCA CCC CAC ATT ATC TTG-3′). The *FCGR3B* alleles and genotypes were determined based on the published reference [16], [17].

**Table 1:** Primers and probes of TaqMan *FCGR* gene SNP assays.

Gene (SNP)	Gene-specific primers (5′ to 3′)	TaqMan Primers and Probes (5′ to 3′)
***FCGR2A***	TGCCATAAGAGAATGCTCACA	CCAGAATGGAAAATCCCAGAAA
(rs1801274)	TCAAAGTGAAACAACAGCCTGACT	TTTGCTTGTGGGATGGAGAAG
		FAM-TCTCCC***G***TTTGGATC
		Vic-TCTCCC***A***TTTGGATCC
***FCGR2B***	CTAAGAGGAGCCCTTCCCTATGT	CCCTAGCTCCCAGCTCTTCA
(rs1050501)	AATACGGGCCTAGATCTGAATGTG	TGCAGTAGATCAAGGCCACTACA
		FAM-TCACTGGGA***C***TGCT
		Vic-CACTGGGA***T***TGCTG
***FCGR2C***	CTGCATATGTTGTCCCCCTGTGTTGCTAAAT	TCAGCAGCTCCCCCAAAG
(rs10917661)	AACATGAGAGAGAAAAAGAGAGGCAGGG-	CGGCATGTCAGAGTCACAGAGT
	AGGGAGCTTA	FAM-AAACTCGAGCCC***C***AGTG
		Vic-CTCGAGCCC***T***AGTGG
***FCGR3A***	CTGGTGTTTACATTGAGTTCTC	AAGACAGCGGCTCCTACTTCTG
(rs396991)	CTGATTCTGGAGGCTGGTTCTACA	GTTCACAGTCTCTGAAGACACATTTTT
		FAM-AGGGGGCTT***G***TT
		Vic-AGGGGGCTT***T***TTG

Italic and underlined nucleotides are SNP sites in respective *FCGR* genes.

### Statistical Analysis

The IgE levels were log-transformed to correct for skewed distribution. Family-based association tests (FBAT) [18] were used to examine whether individual *FCGR* SNPs are associated with phenotypes of human subjects in the asthma family cohort. Alternatively, we used conditional logistic regression to estimate odds ratios of *FCGR* SNPs for their association with asthma, BHR, and atopy, adjusting for age and sex. The association between log-transformed IgE levels and *FCGR* genotypes were analyzed using one-way analysis of variance (ANOVA) in addition to the nonparametric t-test (Mann-Whitney test). In both FBAT and regression analysis, an additive model was assumed for SNP genotypes. To correct for multiple hypothesis tests, the Bonferroni method was used and the null hypothesis was reject at 0.05/number of tests.

## Results

### The FcγRIIA SNP is Associated with Atopy

As shown in Table 2, the *FCGR2A* SNP (FcγRIIA-131His>Arg, rs1801274) is significantly associated with atopy in the family-based association test (FBAT) (*P* = 0.003). The *FCGR2A* SNP is also associated with asthma and BHR in FBAT (*P*<0.05). Conditional logistic regression analysis estimated an OR of 0.732 (*P* = 0.027, 95% CI: 0.555–0.965) for *FCGR2A* SNP with atopy, suggesting a protective role against atopy for carriers of the FcγRIIA-131His allele (population allele frequency = 0.488). Although the *FCGR2A* SNP is significantly associated with asthma and BHR in FBAT (*P*<0.05), the association were not significant in logistic regression analyses adjusted for age and sex. Further validation may be needed to confirm our findings. Furthermore, the functional SNPs of the other three activating FcγRs (FcγRIIIA, FcγRIIIB, and FcγRIIC) were not associated with asthma, BHR, and atopy (*P*>0.05) (Table 3).

**Table 2:** *FCGR2A* and *FCGR2B* SNPs are associated with atopy.

Genes/Traits	FBAT		Logistic regression adjusted for age & sex	
	Z Score	*P*	*P*	OR (95% CI)
*FCGR2A*				
Asthma	2.542	**0.011**	0.187	1.229 (0.906–1.671)
BHR	2.498	**0.012**	0.207	1.214 (0.898–1.642)
** Atopy**	**2.981**	**0.003**	**0.027**	**0.732 (0.555–0.965)**
*FCGR2B*				
Asthma	0.692	0.489	0.476	0.870 (0.594–1.275)
BHR	0.822	0.411	0.906	0.978 (0.676–1.410)
** Atopy**	0.341	0.733	**0.003**	**1.758 (1.209–2.556)**

*FCGR2A* SNP (FcγRIIA-131His>Arg, rs1801274) is significantly associated with atopy in family-based association tests (FBAT). Logistic regression analysis also demonstrated that *FCGR2A* SNP is significantly associated with atopy and that the FcγRIIA-131His (allele frequency: 0.488) is a protective allele against atopy (*P* = 0.027, OR 0.732, 95%CI: 0.555–0.965). The *FCGR2A* SNP is also associated with asthma (*P* = 0.011) and BHR (*P* = 0.012) in FBAT.

The *FCGR2B* SNP (FcγRIIB-187Ile>Thr, rs1050501) is significantly associated with atopy (*P* = 0.003, OR 1.758, 95%CI: 1.209–2.556) in logistic regression analyses adjusted for age and sex. The *FCGR2B* SNP is not associated with asthma and BHR (*P*>0.05).

**Table 3:** Functional SNPs of *FCGR3A, FCGR3B,* and *FCGR2C* are not associated with asthma, BHR, and atopy.

Gene	MAF	Asthma		BHR		Atopy	
		Z	*P*	Z	*P*	Z	*P*
*FCGR3A*	0.378	0.359	0.7194	0.618	0.5363	0.303	0.7615
*FCGR3B*	0.362	0.984	0.3253	1.129	0.2590	1.500	0.1336
*FCGR2C*	0.157	0.159	0.8740	0.141	0.8875	0.563	0.5734

SNPs of *FCGR3A* SNP (FcγRIIIA-158Val>Phe, rs396991), *FCGR3B* allele (FcγRIIIB-NA1/NA2), and *FCGR2C* SNP (FcγRIIC-13Gln>Stop, rs10917661) are not associated with asthma, BHR, and atopy in family-based association test (FBAT) analyses (*P*>0.05) and logistic regression analyses adjusted for age and sex (*P*>0.05, data not listed).

**MAF**: minor allele frequency.

### The Inhibitory FcγRIIB SNP is Associated with Atopy and IgE Production

**Figure 1:**
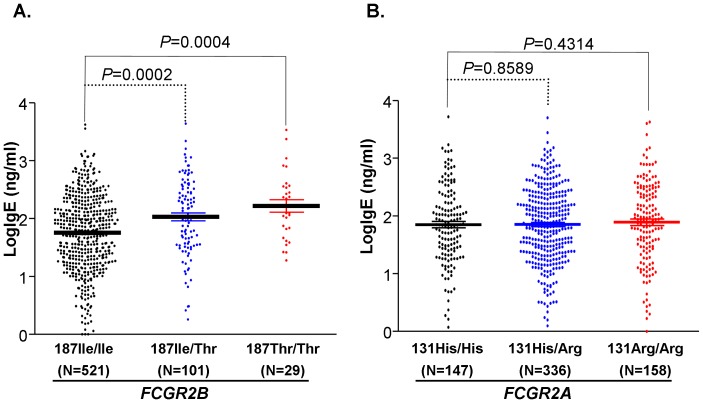
Association of SNP *FCGR2B*-187Ile>Thr with IgE levels. **A.** The genotypes of *FCGR2B*-187Ile>Thr were significantly associated with IgE levels in ANOVA (*P*<0.0001). The rare *FCGR2B*-187Thr carriers (187Ile/Thr and 187Thr/Thr genotypes) produced significantly more IgE than the homozygous subjects for common allele (187Ile) in Mann-Whitney tests. **B.** The genotypes of *FCGR2B*-131His>Arg were not associated with IgE levels in ANOVA (*P* = 0.817). No significant differences were found between *FCGR2A*-131Arg carriers (131his/Arg and 131Arg/Arg genotypes) and the 131His homozygous subjects in Mann-Whitney tests (*P*>0.05).

Although the *FCGR2B* SNP (FcγRIIB-187Ile>Thr, rs1050501) is not associated with asthma, BHR, and atopy in FBAT analyses, conditional logistic regression analyses showed that *FCGR2B* SNP is significantly associated with atopy and that the FcγRIIB-187Thr (allele frequency = 0.088) is a risk allele for atopy (*P* = 0.003, OR 1.758, 95% CI: 1.209–2.556) (Table 2). Because immunoglobulin E (IgE) play an important role in allergic diseases and elevated total IgE is frequently considered as a diagnostic criterion for allergic diseases [3], we subsequently analyzed whether the FcγRIIB SNP is associated with IgE levels in human subjects. As shown in Figure 1A, FcγRIIB genotypes are significantly associated with the serum IgE levels. The human subjects carrying rare FcγRIIB-Thr allele produced significantly more IgE (*P* = 0.0002 for 187Ile/Thr heterozygous subjects and *P* = 0.0004 for 187Thr/Thr homozygous subjects) than those homozygous (187-Ile/Ile) subjects carrying the common allele, suggesting that the functional FcγRIIB SNP may have a role in allergy through IgE production. On the other hand, FcγRIIA SNP is not associated with IgE production in humans (Figure 1B).

## Discussion

This study shows the association of two functional SNPs (the activating FcγRIIA-131His>Arg and the inhibitory FcγRIIB-187Ile>Thr) with human atopy. Furthermore, we demonstrated an association between FcγRIIB SNP and IgE production. Our data indicate a role for IgG Fc receptors in the development of allergy.

FcγRIIA is expressed on the surface of various immune cells including mast cells, basophils, neutrophils, monocytes, dendritic cells, macrophages, and platelets [19], [20]. The FcγRIIA-131His>Arg SNP significantly affects receptor binding affinity and specificity for IgG subclasses [21], [22]. Although both FcγRIIA-131His and 131Arg alleles bind IgG1 and IgG3, the FcγRIIA-131His allele displays a higher binding affinity for IgG3 and is capable of binding IgG2 most effectively as compared to the FcγRIIA-131Arg allele [21], [22]. The functional FcγRIIA-131His>Arg SNP affects the receptor binding affinity for IgG and thus influences the clinical phenotype in response to infectious diseases and inflammation [21]. The FcγRIIA-131His>Arg SNP affects the functions of bacterial phagocytosis [23], [24] and immune complex handling [22], [25], [26]. The FcγRIIA-131His>Arg SNP has been reported to be associated with ulcerative colitis [27], Kawasaki diseases [28], systemic lupus erythematosus [29], and chronic inflammatory disorders such as periodontitis [30], [31] and Guillain-Barré syndrome [32]. In addition, FcγRIIA-131His>Arg polymorphism is associated with infections including recurrent bacterial respiratory tract infections [33], bacteremic pneumococcal pneumonia [34], severe acute respiratory syndrome [35], severe sepsis [36], HIV [37], and EB virus infection [38]. IgG2 is produced primarily in response to polysaccharide/carbohydrate antigens commonly found in allergens. The protective effect of the FcγRIIA-131His allele on atopy is possibly due to the increased capacity of this allele to efficiently internalize and destroy allergen-IgG2 immune complexes. The role of FcγRIIA in allergy was also demonstrated in transgenic mouse models [39]. Therefore, FcγRIIA likely contributes to allergy development in humans. Although the FcγRIIA-131His>Arg SNP is associated with atopy, the SNP is not associated with IgE production (Figure 1B), suggesting that FcγRIIA likely affects allergy through pathways of immune complex clearance and receptor-mediated cell activation. Future studies are required to reveal whether IgG2 levels are associated with the asthma or atopy in the context of FcγRIIA SNP and whether FcγRIIA-mediated functions (immune complex clearance and phagocytosis of allergens) are different between asthmatic and non-asthmatic human subjects.

FcγRIIB, mainly expressed on B cells and myeloid cells, is a classical inhibitory IgG Fc receptor [40], [41], [42]. Cross-linking of FcγRIIB by immune complexes leads to the down-regulation of B cell activation and antibody production, which is an important feedback mechanism to maintain the homeostasis of immune responses [5], [40], [43]. Therefore, FcγRIIB overexpression (or enhanced FcγRIIB functions) reduces the immunoglobulin production in T-dependent immune responses [44]. In humanized mouse models of immunoglobulin production, co-engagement of IgE B-cell receptor with FcγRIIB drastically inhibited human IgE production [45]. FcγRIIB-187Ile>Thr SNP (rs1050501) is located within the receptor transmembrane segment and the FcγRIIB-187Thr allele is less efficient in mediating inhibitory signals than the FcγRIIB-187Ile allele [46], [47], [48]. We observed that the low function FcγRIIB-187Thr allele is significantly associated with elevated IgE levels (Figure 1), suggesting that the reduced FcγRIIB function may promote IgE antibody production by B cells in humans. Interestingly, the low function FcγRIIB-187Thr allele is also associated with protection against malaria [49], signifying FcγRIIB functions play important roles in controlling the immune response to parasites [50]. Nevertheless, FcγRIIB-187Ile>Thr SNP may also be in linkage equilibrium with SNPs of the *FCER1A* gene encoding for the alpha chain of the high affinity receptor for IgE (FcεRIA) because a GWAS identified the *FCER1A* functional variants strongly associated with total IgE levels [51].

FcγRIIB on immune cells also inhibits cellular functions including phagocytosis, ADCC, degranulation, and cytokine release [40]. Mast cells from FcγRIIB^−/−^ mice are highly sensitive to IgG-triggered degranulation compared to those from the wild-type mice. FcγRIIB-deficient mice have an enhanced passive cutaneous analphylaxis reaction, as a result of the decreased threshold for mast-cell activation through activating Fc receptors [52]. FcγRIIB negatively regulates cell activation triggered by high-affinity IgE receptors (FcεRI) [53]. FcγRIIB binds to the Fc domains of IgE and IgG with similar low affinity [54], [55]. Mast cells and basophils could be regulated by immune complexes of allergen–IgG or allergen–IgE. FcγRIIB-deficient mice developed more severe eosinophilia compared to wild-type mice, suggesting an important regulatory role for FcγRIIB in the onset of allergic diseases [56]. FcγRIIB-knockout mice developed the exacerbated lung inflammation [57]. Taken together, FcγRIIB seems to play a critical role in allergic inflammations. In the current study, the dysfunctional FcγRIIB-187Thr allele was found to be a risk factor for atopy. A decreased activation threshold for immune cells carrying FcγRIIB-187Thr allele may be responsible for the increased sensitivity to allergens that trigger the allergic responses, which may explain the association between the defective FcγRIIB allele and atopy.

On the other hand, the functional SNPs of the other three activating FcγRs (FcγRIIIA, FcγRIIIB, and FcγRIIC) were not associated with asthma, BHR, and atopy, suggesting that functions of the restrictively expressed activating FcγRs (FcγRIIIA, FcγRIIIB, and FcγRIIC) may not play prevailing roles in the development of allergy. Our current study had more than 80% power to detect an association between a *FCGR* SNP and atopy with an OR of 1.75.

In summary, the functional SNPs of FcγRIIA and FcγRIIB are associated with atopy, signifying that FcγRIIA and FcγRIIB may serve as important modifiers in the development of allergy. Therefore, targeting FcγRIIA and FcγRIIB for enhanced receptor expressions and functions may be an important avenue for therapeutic discovery in allergy and asthma treatment.

## Supporting Information

Table S1 Distribution of *FCGR2A* SNP (rs1801274) in atopy^+^ and atopy^-^ subjects.
(DOC)

Table S2 Distribution of *FCGR2B* SNP (rs1050501) in atopy^+^ and atopy^-^ subjects.
(DOC)
