# Supplementary material for: Functional Fcgamma Receptor Polymorphisms Are Associated with Human Allergy
Source: PLoS One. 2014 Feb 21;9(2):e89196. doi: 10.1371/journal.pone.0089196 (PMC3931680; doi:10.1371/journal.pone.0089196)
Supplement: Table S1 — Distribution of FCGR2A SNP (rs1801274) in atopy+ and atopy- subjects. (DOC) [file pone.0089196.s001.doc]

Table S1. Distribution of *FCGR2A* SNP (rs1801274) in atopy^+^ and atopy^-^ subjects

|  | **Atopy^+^**  N = 373 | **Atopy^-^**  N = 243 | **χ^2^** | ***P* value** | **OR (95% CI)** |
| --- | --- | --- | --- | --- | --- |
| **Genotype** |  |  |  |  |  |
| AA (%) | 79 (21.2) | 63 (25.9) | 5.921 | 0.0518 |  |
| AG (%) | 191 (51.2) | 133 (54.7) |  |  |  |
| GG (%) | 103 (27.6) | 47 (19.4) |  |  |  |
|  |  |  |  |  |  |
| **Allele frequency** |  |  |  |  |  |
| A (%) | 349 (46.8) | 259 (53.3) | 4.989 | 0.0255 | 1.298 (1.032-1.632) |
| G (%) | 397 (53.3) | 227 (46.7) |  |  |  |

*FCGR2A* SNP rs1801274G allele is significantly associated with atopy (χ^2^ test *P* = 0.0255, OR 1.298, 95%CI: 1.032-1.632).
