# Supplementary material for: Functional Fcgamma Receptor Polymorphisms Are Associated with Human Allergy
Source: PLoS One. 2014 Feb 21;9(2):e89196. doi: 10.1371/journal.pone.0089196 (PMC3931680; doi:10.1371/journal.pone.0089196)
Supplement: Table S2 — Distribution of FCGR2B SNP (rs1050501) in atopy+ and atopy- subjects. (DOC) [file pone.0089196.s002.doc]

Table S2. Distribution of *FCGR2B* SNP (rs1050501) in atopy^+^ and atopy^-^ subjects

|  | **Atopy^+^**  N = 370 | **Atopy^-^**  N = 239 | **χ^2^** | ***P* value** | **OR (95% CI)** |
| --- | --- | --- | --- | --- | --- |
| **Genotype** |  |  |  |  |  |
| TT (%) | 285 (77.0) | 207 (86.6) | 8.980 | 0.0112 |  |
| CT (%) | 64 (17.3) | 26 (10.9) |  |  |  |
| CC (%) | 21 (5.7) | 6 (2.5) |  |  |  |
|  |  |  |  |  |  |
| **Allele frequency** |  |  |  |  |  |
| T (%) | 634 (85.7) | 440 (92.1) | 11.32 | 0.0004 | 1.936 (1.311-2.860) |
| C (%) | 106 (14.3) | 38 (7.9) |  |  |  |

*FCGR2B* SNP rs1050501C allele is significantly associated with atopy (χ^2^ test *P* = 0.0004, OR 1.936, 95%CI: 1.311-2.860).
